# Fidelity in Animal Modeling: Prerequisite for a Mechanistic Research Front Relevant to the Inflammatory Incompetence of Acute Pediatric Malnutrition

**DOI:** 10.3390/ijms17040541

**Published:** 2016-04-11

**Authors:** Bill Woodward

**Affiliations:** Department of Human Health and Nutritional Sciences, University of Guelph, Guelph, ON N1G 2W1, Canada; wwoodwar@uoguelph.ca; Tel.: +1-519-823-5301

**Keywords:** acute malnutrition, protein-energy malnutrition, protein-calorie malnutrition, childhood malnutrition, low-protein diet, caloric restriction, animal model, immune depression, cell-mediated immune depression

## Abstract

Inflammatory incompetence is characteristic of acute pediatric protein-energy malnutrition, but its underlying mechanisms remain obscure. Perhaps substantially because the research front lacks the driving force of a scholarly unifying hypothesis, it is adrift and research activity is declining. A body of animal-based research points to a unifying paradigm, the Tolerance Model, with some potential to offer coherence and a mechanistic impetus to the field. However, reasonable skepticism prevails regarding the relevance of animal models of acute pediatric malnutrition; consequently, the fundamental contributions of the animal-based component of this research front are largely overlooked. Design-related modifications to improve the relevance of animal modeling in this research front include, most notably, prioritizing essential features of pediatric malnutrition pathology rather than dietary minutiae specific to infants and children, selecting windows of experimental animal development that correspond to targeted stages of pediatric immunological ontogeny, and controlling for ontogeny-related confounders. In addition, important opportunities are presented by newer tools including the immunologically humanized mouse and outbred stocks exhibiting a magnitude of genetic heterogeneity comparable to that of human populations. Sound animal modeling is within our grasp to stimulate and support a mechanistic research front relevant to the immunological problems that accompany acute pediatric malnutrition.

## 1. Defining the Problem

According to a recent estimate [1] nearly 7,000,000 children lose their lives annually before the age of five years and, of this number, 800,000 expire pursuant to acute deficits of dietary protein and energy (*i.e.*, deficits causing wasting and/or edema) acting in synergy with infectious diseases. Moreover, this toll of mortality undoubtedly is exceeded numerically by an additional burden of infection-related morbidity. Immune depression is widely accepted as an important link between acute forms of malnutrition and susceptibility to infection [2,3], and repairing inflammatory immune competence has been identified as one of three preferred, complementary approaches to reducing the burden of infection in acute forms of pediatric protein and energy deficit [4]. In fact, immune restoration may be the only viable strategy for the most severely wasted patients whose infections will otherwise overwhelm them before their weight loss can be addressed [5]. Interventions of this nature, however, clearly require a sophisticated grasp of malnutrition-associated immunopathology.

A recent review of literature pertaining to human subjects [3] demonstrates that, despite sixty years of research activity, our grasp of immune competence in pediatric malnutrition has failed to advance beyond the stage of cataloging descriptive immunological phenomena. Moreover, after an early flurry of investigative activity that peaked around 1980, research effort (judged in terms of numbers of publications) has fallen steadily and precipitously to the present day [3]. Sadly, this is not surprising. The research front is adrift, lacking a unifying paradigm with sufficient scholarly energy to provide form, coherence, or direction. If the field can be said to possess a unifying hypothesis at all, it is the longstanding and largely unspoken proposition that malnutrition-associated immune depression reflects a chaotic across-the-board immunological attrition [2]. This simplistic model was sufficient to stimulate important early descriptive studies, but it lacks the sophistication required to propel a mature, mechanistic research front. In short, the field is dying a death of intellectual marasmus, having embraced no fundamentally new thinking since its inception decades ago and clinging to a canon, if to any central tenet at all, that has outlived its useful life [2].

Rytter *et al.* [3] concluded their very helpful state-of-the-art assessment by outlining some mechanistic ideas pertaining to malnutrition-associated immune depression and by providing some suggestions regarding future research directions. These concluding remarks, particularly the research suggestions, are revealing in their almost exclusive focus on studies of human subjects—an accurate representation of the prevailing point of view within the field that studies of humans are required to generate findings relevant to humans and that animal-based investigations, if encouraged at all, should be assigned nothing more than adjunctive status. That said, over a period of at least three decades, a growing cluster of loosely-knit animal-based investigations has proven increasingly difficult to reconcile with the model of chaotic immunological attrition pointing instead toward a much-needed paradigm shift within the field [2,6]. Hence, it may be instructive to identify practices, points of incautious extrapolation, or even points of intellectual neglect that continue to give rise to doubt regarding the relevance of the animal models used to investigate the inflammatory immune depression of acute pediatric malnutrition. The main objective in so doing is to assess the reasonable potential of animal modeling to stimulate a viable and relevant mechanistic research front.

## 2. Modeling Considerations with a View to Improving the Relevance of Animal-Based Investigations Pertaining to Immune Competence in Acute Pediatric Malnutrition

To date, animal modeling of acute pediatric malnutrition has been practiced in a laissez-faire manner apart from broad ethical limits set by bodies that govern the funding, conduct and publication of research. Certainly no broadly based attempt has been made within the research front to establish standards regarding the fidelity with which animal models duplicate human malnutrition pathologies. Consequently, unresolved controversies and prevalent design weaknesses persist, animal modeling is demonstrably crude, and the numerous and diverse models in the field are generally difficult both to relate to one another and to connect with any particular form of pediatric malnutrition. A brief consideration follows of some major modeling characteristics that currently limit the relevance of animal-based studies intended to probe the immunopathology of pediatric malnutrition. Each of the modeling limitations and controversies highlighted here should be amenable to formal resolution.

### 2.1. Model a Human Diet or a Human Pathology?

Human malnutrition pathologies are categorized according to standards centered on weight, height and chronological age. No comparable standards have been established for experimental animals, although a preliminary attempt with one strain of mouse is noteworthy [7]. Taking another approach, one research group applied weight deficit cutoffs devised for children to a suckling rat model of acute malnutrition [8], although the rigor of this direct and simple cross-species strategy remains undefined. The key point is that, at this time, few experimental malnutrition pathologies can be connected with precision to any particular form, or degree, of human malnutrition. By contrast, dietary composition is easily determined for the purpose of comparisons across species and, as a direct result, much skepticism regarding the value of animal models of acute pediatric malnutrition centers on details of dietary formulation. An essential component of modeling fidelity, however, is clarity regarding exactly what is to be modeled. For example, if an animal protocol is intended to shed light on human edematous malnutrition, it is essential to produce features of either incipient or full-blown kwashiorkor, or marasmic kwashiorkor, but arguably less important to duplicate details of a human diet associated with these pathologies. To illustrate this point, a consideration of animal models of protein deficiency follows.

Muroid rodents have been used extensively, and rabbits occasionally, in studies pertaining to acute pre-pubescent malnutrition and anti-infectious defences. It is not surprising to discover differences between humans and these coprophagous species regarding the details of dietary nitrogen content required to produce an edematous pathology. In fact, weanling rodent formulations matching the nitrogen content of diets associated with childhood kwashiorkor often produce, instead, a stunting disease (e.g., [9,10]) that is irrelevant not only to edematous malnutrition [11] but also to stunting malnutrition as this pathology presents in human populations. Rodent stunting models frequently elicit increases in the vigor of inflammatory cell-mediated responses and their corresponding infectious disease resistance [10,12], an outcome in stark incongruity with the immunological and disease resistance characteristics of both stunting pediatric malnutrition and acute malnutrition in children and weanling rodents [2,13]. Even in the case of a mouse model of nitrogen deficit stunting that depressed resistance to infectious disease (albeit a type of resistance independent of inflammatory responses) [14], the model failed to reflect the pathology of pediatric malnutrition in essential respects. In fact, the low-protein stunting rodent model has provided no unambiguous insight into the longstanding epidemiological observation [13,15] that pediatric malnutrition, regardless of form or degree, increases the risk of infection-related mortality in childhood.

It would be shortsighted to dismiss the potential of the low-protein stunting model as a laboratory tool with which to probe immunological plasticity, but focusing on a detail of dietary composition rather than on details of a pathophysiology has produced a type of animal model bearing no clear relationship to any human pathology. By contrast with stunting models, appropriately crafted protocols that produce a negative nitrogen balance in rodents, albeit by means of dietary nitrogen levels uncharacteristic of human consumption patterns, nevertheless reproduce the diagnostic features of kwashiorkor in weanlings (e.g., [16]) and produce the depressed inflammatory immune competence (e.g., [9,17,18,19,20,21,22,23]) and susceptibility to opportunistic and other infections [17,19,21,23,24,25,26,27] that characterize the human pathology.

Endocrinological considerations lead to the same conclusion regarding low-protein weanling rodent models, a point discussed elsewhere [11] in relation to the blood thyroid hormone profile. For many years it was widely held that acutely malnourished rodents do not mimic the thyroid hormone response seen in acute pediatric malnutrition [11]. However, when the focus of attention was shifted from minutiae of dietary composition to reproducing critical features of the pathology under investigation, the resulting rodent models proved satisfactory [11]. Animal modeling for the purpose of probing the pathology of acute malnutrition is first and foremost an exercise in physiology, not in dietetics.

This is a significant and controversial matter that extends far beyond the research front under consideration here. For example a prestigious international journal centered on basic nutritional science has recently adopted the policy that “Studies involving animal models of human nutrition and health or disease will only be considered for publication if the amount of a nutrient or combination of nutrients used could reasonably be expected to be achieved in the human population” [28]. Clarity of thought regarding the core essence of animal modeling fails with surprising frequency and on a surprisingly grand scale.

### 2.2. Genetic Diversity

The limited genetic diversity of animals used in modeling acute malnutrition, when compared to the diversity within and among human populations, is a longstanding and important concern. In particular, inbred strains of mice have been widely used for immunological studies because of the powerful array of available reagents. Panels of inbred strains can be used for the purpose of testing against a broader genetic background [29], although full application of this strategy is cumbersome. In the context of modeling the immunopathology of pediatric malnutrition, a much truncated version of this type of protocol has been applied by one research group which conducted simultaneous parallel studies with two distantly-related inbred mouse strains [30,31,32,33]. Use of such an abbreviated panel may reduce the risk of being led astray by an idiosyncratic strain-specific response, but it can accomplish little more.

Outbred strains offer the advantage of heterozygosity in which each animal is genetically unique, and examples of the use of outbred animals in modeling acute pediatric malnutrition are numerous. That said, the genetic diversity of standard outbred strains of mice does not model a fully outbred population and, hence, is limited relative to that of most human populations [34,35]. The Diversity Outbred strain of mouse was developed in response to this shortcoming [36], and the Collaborative Cross population of mice is a recombinant inbred resource developed with the same intent [37]. To date, however, attempts to model acute pediatric malnutrition against a background of meaningful genetic heterogeneity have not extended beyond the use of conventional outbred strains of rodents or, occasionally, rabbits.

It is important to acknowledge that many features of the immunopathology of acute pediatric malnutrition have been reproduced against diverse inbred and outbred genetic backgrounds and have been duplicated, also, across species. However, advancement beyond a largely descriptive phase toward a meaningful pursuit of mechanisms deserves a modeling strategy designed to account for the unknown scope and impact of a magnitude of genetic diversity comparable to that typical of human populations.

### 2.3. Dietary Strategies

Two basic dietary strategies predominate in animal modeling of acute protein and energy deficits, viz. restricted intake of a complete diet and ad libitum consumption of an imbalanced diet containing a low level of nitrogen relative to calories (but complete in all other known respects). Complete diets for rodents are sufficiently nitrogen-rich that, apart from the occasional strategy of acute withdrawal of all nutrients except water, a regimen sometimes imposed on adult animals (e.g., [38,39]) but not on weanlings, even the most extreme restricted intake protocol elicits a caloric deficit without imposing a deficiency of protein (e.g., [40,41]). By contrast, some low-protein diet protocols reduce food consumption sufficiently to induce a concomitant caloric deficit (e.g., [22,40,41,42]), whereas others appear to produce an exclusive nitrogen insufficiency (e.g., [11,16,21,43]). Occasionally, restricted intake of a nitrogen-deficient diet is imposed to ensure a combined deficit of both protein and calories [7,44,45].

Regardless of the basic type of model, effort is usually made to ensure that dietary levels of micronutrients at least meet the species-specific standards of a complete diet. In this respect, the diet formulations usually used in studies of experimental malnutrition differ from the micronutrient-poor diets associated with malnutrition in childhood [46], although an occasional exception can be cited. Notably, one group has developed a protocol involving restricted intake of a diet deficient in iron and zinc as well as in nitrogen (e.g., [7,44,45]). The real importance of this factor remains unclear, however, given that apparent dietary micronutrient sufficiency does not guarantee metabolic micronutrient sufficiency in animal models of malnutrition (e.g., [9,47]). Further, the restricted intake type of experimental protocol is never preceded by a period of stunting malnutrition, whereas pediatric marasmus, which a restricted intake protocol is intended to reproduce, is commonly superimposed on an established stunting disease [48]. Despite these considerations, the restricted intake type of model can duplicate key features of pediatric marasmus and the low-protein type of model can reproduce hallmark features of incipient and full-blown kwashiorkor [49]. The significance of the disparities in dietary backdrop between pediatric malnutrition and its animal models deserves assessment as part of any larger initiative aimed at the relevance of animal-based experimentation.

### 2.4. Species-Specific Immunological Characteristics

Muroid rodents have been the most widely used experimental animals in studies pertaining to malnutrition and anti-infectious defences. This undoubtedly reflects the arsenal of reagents that has been available, and growing, for decades to probe the immunological defences of these species, particularly the mouse. Despite substantive broad similarities between the immune defences of humans and muroid rodents, however, there are a great many differences in points of detail as outlined elsewhere in relation to the laboratory mouse [50,51]. These differences can represent important species-specific distinctions in the biology of infectious disease resistance, distinctions that are only magnified by the use of inbred animals [51]. The “humanized” mouse, produced by seeding immunodeficient animals with human hematopoietic stem cells, is beginning to extend the reach of animal models to achieve something akin to studies of human anti-infectious immune responses *in vivo* [52,53]. This technology, however, has not yet been applied to investigations of the connection between malnutrition and susceptibility to infection. Hence, the contribution of animal models to our knowledge in this field derives entirely from the use of conventional animal systems. Two examples based on published animal modeling of the immunopathology of acute pediatric malnutrition will illustrate the need to respect species differences from humans in the application of conventional animal models.

(a) Secretory IgA is released into the intestinal mucus of humans mainly by transport across the intestinal epithelium, whereas the liver of humans plays a negligible role in this function [54]. Diverse species including guinea pigs, dogs, sheep and nonhuman primates resemble humans in this respect; by contrast the liver plays a dominant role in transporting IgA to the intestinal lumen of other species including rats, mice, hamsters, rabbits and chickens [54]. Hence, the latter species present an inferior opportunity to model humans with respect to the impact of acute malnutrition on non-inflammatory mucosal blocking antibody defence, although both the intestinal epithelium and the liver may play important roles in the mouse [55]. Relatively little research attention has been given to mucosal immune competence in acute malnutrition despite the prevalence of opportunistic mucosal infections among the malnourished [3,13]. Muroid rodent models of dietary protein and energy deficit have been of some value by directing attention to the IgA receptor as a point of particular interest in relation to the mucosal infections that characterize pediatric protein and energy deficits [56,57,58]. Nevertheless, relevant animal-based investigations will require a shift of attention away from rats and mice to more appropriate animal species.

(b) Invasive procedures are required to access macrophages in sufficient numbers for research purposes. For this reason most information pertaining to malnutrition and the mononuclear phagocyte system, with the exception of the blood monocyte, derives from studies of experimental animals, [13]. In turn, the species most commonly selected have been rats and mice [13]. The microbicidal activity of macrophages from muroid rodents depends, in significant measure, on reactive nitrogen species whereas, although a matter of controversy [50], this mechanism is widely considered unlikely to be important in macrophages of humans [59]. In any case, even if the inducible nitric oxide synthase system plays a part in the macrophages of humans, it is activated by an entirely different constellation of stimuli from the family of mediators found effective in rats and mice [50]. Guinea pigs [60] and rabbits, pigs and goats [59] may resemble humans more closely than do rats and mice with respect to the microbicidal arsenal of the macrophage, and the guinea pig has been used to advantage in a series of studies pertaining to pulmonary tuberculosis in acute malnutrition [17,61]. However, the full extent of the similarities and differences between various species and humans in this regard remains to be determined. A technology such as the humanized mouse [52,53] may provide a more productive strategy with respect to this type of modeling problem.

### 2.5. Modeling According to Stage of Immunological Ontogeny

Five stages of mammalian immunological ontogeny are commonly recognized [62]. A consequence of this developmental characteristic is that the impact of an external insult on immune competence depends on the ontogenetic stage, or so-called “window of vulnerability”, during which the insult is imposed [62]. Although the five immunological windows of vulnerability are common to mammalian species, the timing of their appearance relative to other developmental milestones varies among species; in addition, as must be expected, the duration of each immunological window is also species-specific [62]. For example, the capacity for adaptive immune memory (a component of the fifth stage of ontogeny) emerges in association with full-term parturition in humans but generally develops after weaning in rats and mice [62], although common inbred mouse strains are reported to differ dramatically in this respect [63].

The importance of immunological ontogeny when attempting to model the immunopathology of pediatric malnutrition can be illustrated by means of a small cluster of reports involving two inbred mouse strains [9,18,43,64,65]. In this series of investigations, a primary antibody response (associated with the fourth stage of immunological ontogeny) was assessed *in vivo* in weanlings of both strains at the same chronological age following subjection to a standardized protocol of acute protein deficit. The response was profoundly depressed in the weanlings of the less ontogenetically advanced CBA/J strain [9,64], but was unaffected in the more advanced C57BL/6J strain [43]. Initiating malnutrition only four days earlier during the peri-weaning period, however, appeared to access the desired window of vulnerability in the C57BL/6J strain, eliciting the classic depressive influence on primary humoral competence [18,65].

The decisive importance of immunological windows continues to escape notice in animal modeling of the immunopathology of pediatric malnutrition. For example, the potential of the suckling rat model has been outlined recently in convincing detail for the purpose of studies pertaining to the influences of pediatric nutrition on immunological development and competence [66], yet the factor of immunological windows of vulnerability evaded explicit attention. The suckling period covers stages of immunological development in the rat that are completed during a normal full term gestation in the human [62]. Hence, the suckling rodent model can be used profitably to study phenomena relating to immunological imprinting [66] but its value is obscured—and wider opinion of the relevance of animal modeling is not enhanced—by the suggestion (e.g., [8,66]) that a suckling model sheds light regarding the immunological impact of malnutrition initiated during infancy or childhood. Some weanling animal models used to date undoubtedly have captured an immunological window relevant to the pediatric stages of life, but a mature research front will require purposeful validation of models according to this decisive factor.

### 2.6. Zero-Time Control

Weanling models of acute malnutrition present the risk that diet-related influences may be confounded by ontogeny. Consequently a zero-time control has been used occasionally when modeling acute pediatric malnutrition-associated immune depression (e.g., [16,40,49,56,67,68]). As the following examples illustrate, the risk of propagating misinterpretations and oversights can be high in the absence of this rare design.

(a) Comparison with an age-matched control group emphasized the small sizes of both the splenic IgG-containing cellular compartment and the intestinal IgA-containing cellular compartment of acutely malnourished weanling mice [16]. Many comparable observations can be found in the published information base [13], and the widely-held interpretation, essentially unchallenged, has been that these findings reflect compartmental atrophy. However, in the cited report, comparison with a zero-time control group revealed that the well-known expansion in splenic and intestinal plasma cell numbers during the weanling stage of life was sustained, but at an attenuated rate [16]. Without inclusion of a zero-time control group in the design of this work, the robustness of weanling-stage antibody-producing effector compartments in the face of catabolic malnutrition would have remained hidden.

(b) Among the most striking immunological features of acute pediatric malnutrition is the small size and denuded architecture of T cell compartments in secondary lymphoid organs [13,69]. This consistent observation has been widely presumed to reflect a process of attrition, rather than simply a failure of normal ontogenetic increase, and studies of weanling mouse models utilizing a zero-time control design [32,33,70,71] have confirmed this reasonable expectation. However, use of this design feature also revealed that attrition takes place predominantly within subpopulations of T cells exhibiting an effector/memory phenotype [33,70,71], perhaps exclusively so within the CD4^+^ subset [70]. In turn, the unexpected robustness of naïve-phenotype T cell populations in the face of catabolic malnutrition gave rise to studies of dendritic cells because of the unique potency of these antigen presenting elements for activating the naïve T cell. Ultimately, this pursuit generated a challenge to the classic T cell-centric focus of the research front by placing the dendritic cell and antigen presentation at the center of malnutrition-associated inflammatory immune depression [22,72].

(c) Acutely malnourished weanling mice exhibit some immunological characteristics that resemble neonatal forms of competence. For example, a type 2 cytokine polarization is reported on the part of both the effector/memory T cell compartment [68] and the blood cytokine profile [40] of weanlings subjected to metabolically diverse forms of acute malnutrition. Inclusion of a zero-time control in these investigations permitted the conclusion that these features are part of a regulated, systemic restructuring toward a non-inflammatory form of immune competence, the antithesis of a chaotic descent into immunological incompetence and a profound adaptive attempt rather than a biologically trivial delay in ontogeny [2,68].

## 3. Systemic and Cellular Immunobiology of Acute Pediatric Malnutrition: Conceptual Revisions and Clinical Implications Attributable to Animal Modeling

To date, animal modeling of acute pediatric protein and energy deficits clearly leaves much to be desired, and skepticism regarding its real value is understandable. Nevertheless, attention to a modest shortlist of critical design features, notably prioritizing characteristics of human pathology rather than dietary minutiae specific to humans, recognizing the decisive importance of immunological ontogeny, and use of a zero-time control, has permitted some animal-based research to yield insights unavailable from clinical or epidemiological investigations. Arguably some uniquely animal-derived contributions can be characterized as sufficiently far-reaching and fundamental that, considered together, they provide a foundation for a unifying hypothesis to probe the mechanistic underpinning of malnutrition-associated immunopathology. The following commentary, elements of which can be found elsewhere [2,6,13,69], identifies some of these key insights and their significance.

### 3.1. Some Fundamental Insights

Malnutrition-induced depression in immune defences and infectious disease resistance is often portrayed as consequent to a disintegration of metabolic control, most notably a non-selective and systemic decline in protein synthesis [2,13,69]. However, a small cohort of animal-based findings, some of which are summarized elsewhere [2,13,69], mounts an insistent challenge to this point of view, and a few reports from studies of human subjects are readily interpreted as consistent with this challenge [3], at least as a point of logic. For example, fine control of hepatic protein synthesis is reported in the weanling rat in the face of acute dietary deficits of either nitrogen or calories [73,74]. In other work, an organ-specific (*i.e.*, not system-wide) decline in synthesis of inflammatory cytokines is reported in a rodent model of acute nitrogen deficiency [75]. Further, while the systemic synthesis of inflammatory cytokines declines, the synthesis of key non-inflammatory cytokines is either sustained or even increased in the face of advanced weight loss in metabolically distinct models of weanling malnutrition [41,68,76,77]. Collectively, these findings from diverse weanling models force at least the suspicion that immune depression in acute malnutrition develops within a context of uninterrupted control over protein synthesis. Other animal-based findings appear broadly consistent with this proposition. In particular, weanling ontogeny is reported to proceed on the part of at least some adaptive immune defence components and functions despite profound weight loss and lymphoid involution [16,49], an outcome interpretable as indirect evidence of persistent fine control over protein synthesis. Evidence that control is sustained over a metabolic process as fundamental as protein synthesis provides a basis for optimism regarding the clinical management of acute malnutrition and, in turn, provides justification for pursuing the mechanisms underlying malnutrition-associated immune depression.

Intervention studies involving metabolically diverse forms of acute, experimental malnutrition reveal that infectious disease resistance and components of both innate and adaptive inflammatory immune competence can be sustained, or even restored, independently of ongoing weight loss and in the face of profound lymphoid involution [20,25,38,39,42,78] even at the weanling stage of life [18,22,31,64,72,79,80]. With two exceptions, one an intervention with a bacterial culture condensate [78] and the other an adoptive transfer design [22], these studies demonstrate responsiveness to endocrine hormones and cytokines on the part of immunological elements and functions, *in vivo*, even during advanced stages of experimental malnutrition pathology. Importantly, the studies span three endocrine hormones (the glucocorticoids, leptin and triiodothyronine) and four hematopoietic cytokines (granulocyte, macrophage and granulocyte-macrophage colony-stimulating factors and fms-like tyrosine kinase 3 ligand), seven independent research groups and eight distinct rodent models of acute malnutrition. None of the experimental systems used in these investigations could be characterized as sophisticated, but the weight and diversity of evidence is undeniable. An overriding implication is that rapid enhancement of infectious disease resistance can be achieved even without prior stabilization of severely malnourished patients. This realization could be seen as sufficient to justify application of research resources toward an understanding of the mechanisms underlying malnutrition-associated inflammatory immune depression.

The animal-based findings cited herein demonstrate manipulation of immune competence independently of nutritional status even in the advanced stages of catabolic malnutrition, and provide proof-of-concept that immune regulation by way of soluble mediators continues uninterrupted in the face of acute forms of pediatric malnutrition. At this stage, the real value of this body of information is in its incompatibility with the classic and prevalent point of view that malnutrition-associated depression in immune competence reflects a chaotic attrition of inflammatory capacities. Collectively, the findings have given rise to an alternative unifying hypothesis dubbed the Tolerance Model [2,41,77].

### 3.2. A Unifying Hypothesis and Hints of a Cellular Mechanism

The Tolerance Model is an animal-based challenge to the classic attritional paradigm and centers on the proposition that acute malnutrition elicits a regulated immunological revision toward a non-inflammatory form of competence that, even in pediatric stages of life, is sustained into advanced stages of wasting disease. The proposed immunomodulation is presented in the model as an attempt to adapt to the catabolic pathology of acute malnutrition, and a presumptive benefit is reduction in the risk of inflammatory autoimmune pathologies albeit at the cost of susceptibility to opportunistic infection. The model is far from static and can accommodate new information pertaining to immune regulation as it comes available. For example, the impact of short-term caloric withdrawal on B cell development appears to be mediated in significant measure through the central nervous system [39], an intriguing pathway of control easily included in the Tolerance Model. Most importantly, the proposition is testable and accommodates a body of experimental information that falls outside the purview of the notion of malnutrition-induced immunological attrition. This, alone, should be sufficient to stimulate renewed interest in the subject. Moreover, a proposition of sustained and uninterrupted immune regulation presents hope for clinical management in the form of a previously unrecognized window of therapeutic opportunity.

As to hints of mechanism, one model of acute weanling malnutrition has been used in studies pointing to the dendritic cell as the primary limiting factor in the depressed adaptive cell-mediated immune competence that consistently accompanies acute pre-pubescent malnutrition [22,72]. Another interesting clue centers on the regulatory T cell compartment which, in the face of catabolic malnutrition, may be sustained in preference to the effector T cell compartment according to evidence from both an adult rodent starvation model [81] and weanling mouse models of marasmus and incipient kwashiorkor [41]. These findings divert attention from the effector T cell-centric focus which is at the core of the model of chaotic attrition, and highlight potentially worthy therapeutic targets from the plethora of immunological components affected by malnutrition.

Despite obvious inadequacies, animal modeling has already challenged understanding of malnutrition-associated immunopathology in a fundamental way. In so doing, animal-based studies have provided some justification, together with specific possibilities, for pursuing mechanistic studies. Substantively improved modeling sophistication will be needed to proceed further, but one might hope to see research strategies that incorporate the design precautions outlined here in studies utilizing the immunological humanizing technology [52,53] and animals drawn from populations exhibiting a magnitude of genetic heterogeneity comparable to that of humans (e.g., [36,37]). Animal modeling performed at this level of sophistication should be expected to yield data sets persuasively relevant to the immunopathologies of pediatric malnutrition.

## 4. Concluding Comment

Twenty-five years ago, while summarizing an international conference under the sponsorship of the World Health Organization, the late Nevin Scrimshaw pointed out “There is still a large gap between what can be demonstrated by experimental single nutrient deficiencies in inbred strains of laboratory animals and evidence of relevance to the phenomenon of increased infection among malnourished human populations. It is still uncertain whether some experimental observations of this kind have any relevance at all to public health nutrition.” [82]. This insight prompted two appeals, made on the international stage, to establish rigorous standards of fidelity in animal models of acute pediatric malnutrition. The first appeal was made to the Fourth International Biennial Conference on Nutrition and Health Promotion (1997) which was jointly sponsored by the International Life Sciences Institute, the American Cancer Society, the U.S. Centers for Disease Control and Prevention, and the Emory University School of Medicine [69]; the second call was made to the 45th Nestle Nutrition Workshop which convened in 1999 [13]. In both instances the thrust of the message was that improved animal modeling is a prerequisite to relevant mechanistic studies, and the implication was that the research front cannot mature in the absence of a robust animal-based component. Since that time isolated attempts, some highlighted herein, have been made to address the problem of modeling deficiencies. Moreover, a disperse literature can be cited reporting research initiatives conducted with a mechanistic intent (e.g., [2,3,6,7,13,23,38,39,41,67,81,83]). However, there has been no broadly-based or concerted effort across the full research front to raise the intellectual bar with respect to animal modeling; hence, the power of animal models to contribute much-needed scholarly depth to the field remains largely unrealized and unrecognized. Nevertheless, as shown here, a testable unifying hypothesis is available based on studies using animal models, and the predictions of this proposition could be pursued to advantage by giving attention to a modest list of design improvements while incorporating some newer laboratory animal technologies.
